# Xeno-free serum replacement for ex vivo culture and expansion of T cells

**DOI:** 10.1186/2051-1426-2-S3-P37

**Published:** 2014-11-06

**Authors:** Karoline W Schjetne, Grethe Økern, Sandra Kuligowski, Tanja Aarvak

**Affiliations:** 1 Thermo Fisher Scientific, Oslo, Norway; 2 Thermo Fisher Scientific, Grand Island, United States

## Background

The manufacture of a majority of clinical T cell products for immunotherapy applications requires ex vivo T cell culture and expansion. Commercialization of T cell manufacturing processes requires reagents that meet regulatory guidelines and ultimately help reduce manufacturing cost of goods. A key component in many T cell culture protocols is human serum, which is expensive and may require testing prior to use for the manufacture of a cGMP-compliant T cell product. To this end, we have developed a xeno-free serum replacement supplement with defined components that can be used in combination with several different cell culture media to support ex vivo expansion of T cells.

## Results

T cells activated ex vivo and expanded with Dynabeads^® ^CD3/CD28 CTSTM and cultured in OpTmizer™ CTS™, X-VivoTM 15, or AIM-V^® ^CTS™ supplemented with pooled human serum or serum free T cell serum replacement showed similar growth kinetics, total fold expansion and transduction efficiency after 2 weeks in culture. Numbers of expanded CD4+ and CD8+ T cells were comparable in the expanded cultures regardless in the presence of human serum or the newly developed SRS-XF. Restimulated T cells expanded in serum free T cell serum replacement show similar cytokine profile and proliferation as T cells expanded in human serum.

## Conclusion

This study shows that human serum may be replaced by a xeno-free formulation in several commonly used cell culture media to support ex vivo expansion and lentiviral transduction of polyclonal T cells. Culturing T cells in serum free T cell serum replacement facilitates a favourable culture profile and immune function. The serum free T cell serum replacement contains only fully tested human-derived or human recombinant proteins which facilitates supply security for clinical large scale and commercial therapies.

